# Real-world evidence reported for clinical efficacy evaluation in European Public Assessment Reports of authorised targeted therapies for solid malignancies: a comprehensive review (2018-2022)

**DOI:** 10.1016/j.esmorw.2024.100039

**Published:** 2024-06-03

**Authors:** J.W.G. Derksen, D. Martins-Branco, A. Valachis, A. Pellat, S.C.M.W. van Nassau, A. Aggarwal, G. Pentheroudakis, M. Koopman, L. Castelo-Branco, S. Delaloge

**Affiliations:** 1 Julius Center for Health Sciences and Primary Care, Department of Epidemiology and Health Economics, University Medical Center Utrecht, Utrecht University, Utrecht, The Netherlands; 2 Scientific and Medical Division, European Society for Medical Oncology, Lugano, Switzerland; 3 Department of Oncology, Faculty of Medicine and Health, Örebro University Hospital, Örebro University, Örebro, Sweden; 4 Department of Gastroenterology and Digestive Oncology Unit, Cochin Hospital AP-HP, Paris, France; 5 Department of Medical Oncology, University Medical Center Utrecht, Utrecht University, Utrecht, The Netherlands; 6 Institute of Cancer Policy, School of Cancer Sciences, King’s College London, London; 7 Department of Health Services Research and Policy, London School of Hygiene & Tropical Medicine, London, UK; 8 Oncology Institute of Southern Switzerland, EOC, Bellinzona, Switzerland; 9 Department of Cancer Medicine, Gustave Roussy, Villejuif, France

**Keywords:** real-world evidence, targeted therapy, oncology, cancer, European medicines agency, marketing authorisation

## Abstract

**Background:**

The role of real-world evidence (RWE) for clinical efficacy regulatory evaluation remains unclear. We aimed to assess and describe the reported use of RWE for clinical efficacy evaluation of authorised targeted therapies for treatment of solid malignancies in Europe.

**Design:**

We studied all authorised indications of targeted therapies for the treatment of solid malignancies granted by the European Medicines Agency between 2018 and 2022. Data were retrieved in March 2023 from European Public Assessment Reports (EPARs). We evaluated the frequency of RWE use for clinical efficacy evaluation and its role based on the reported information in the EPAR, and assessed characteristics and risk of bias of published studies.

**Results:**

Out of 75 authorised indications identified, most related to the treatment of patients with lung (21.3%) or breast (20.0%) cacer, and to advanced settings (89.3%). The use of RWE for clinical efficacy evaluation was reported in the EPAR of 16 (21.3%) indications, tending to increase overtime (15.0%-35.7% in 2018-2022). RWE was more frequently considered in lung (37.5%) and breast (33.3%) cancer indications, for antibody–drug conjugates (60.0%), and conditional approvals (46.7%). We classified RWE’s role as ‘supportive’ confirmatory evidence in 12 of 16 (75.0%) indications. RWE studies were mostly analytical (57.1%), non-international (92.9%), retrospective cohort studies (57.1%), and originated from the United States (78.6%). High or serious risk of bias was identified in different domains of most studies assessed.

**Conclusions:**

RWE was reported to be used for clinical efficacy regulatory evaluation in 21% of targeted therapy indications for solid malignancies, with an increasing trend over time.

## Highlights

• Real-world evidence use for clinical efficacy is sparse in regulatory evaluation.
• We describe its use in authorisation of targeted therapies for solid malignancies.
• We investigated the reported use of RWE in European Public Assessment Reports.
• RWE use was reported in 21% of indications between 2018 and 2022, increasing over time.
• We classified RWE as ‘supportive’ in 75% of EPARs reporting RWE of clinical efficacy.

## Introduction

Evidence of clinical efficacy to support the benefit–risk assessment and authorisation of new therapeutic indications is traditionally generated from randomised clinical trials (RCTs). Evidence from RCTs, however, may be poorly generalisable due to limitations such as strictly selected study populations, distinct from ‘real-world’ conditions.^1^^,^^2^ The existing disparity between study populations participating in RCTs and the real-world patient population that will receive the investigated treatment has resulted in a shortage of information for many patient subgroups not equitably included in trials.^3^^,^^4^ In certain settings, interventional clinical trials are more difficult to conduct (e.g. rare diseases), or unethical (e.g. disease with very poor prognosis and no effective approved therapy for control arm). In these situations, evidence from other sources than traditional clinical trials are required.^5^

With the growing use of real-world data (RWD) to generate real-world evidence (RWE)^6^ and the increased understanding of RWE importance to assess medicines’ performance, both the United States Food and Drug Administration (FDA) and the European Medicines Agency (EMA) recently recognised the complementary role of RWE in regulatory decision making.^7^^,^^8^ For instance, in June 2023,n EMA published an RWE framework to support European Union (EU) regulatory decision making in which they state that ‘more effort is needed to better anticipate the need for such studies and to speed up their initiation to ensure that regulators have access to RWE in a timely manner’.^9^ Moreover, EMA and the European Medicines Regulatory Network are working towards developing a sustainable framework that will streamline the use and establish the value of RWE across the entire medicine’s regulatory lifecycle.

To date, several studies have shown the integration of RWE in either FDA or EMA marketing authorisation applications over the past years,^10^, ^11^, ^12^, ^13^, ^14^, ^15^, ^16^ and new oncology medicines have recently been authorised using RWE for regulatory decision.^13^^,^^14^^,^^16^ The role of RWE has been well recognised in several domains, such as understanding the determinants of variation in care, disease epidemiology and post-marketing safety monitoring. Regarding the latter, observational studies have long been used by EMA’s Pharmacovigilance Risk Assessment Committee to identify, characterise or quantify the post-authorisation safety hazard, confirm the safety profile of a medicine, or measure the effectiveness of risk-management measures. To date, however, the use of RWE has been less established for demonstrating clinical efficacy or effectiveness.

Given the growing importance of precision oncology in personalising oncology treatment, we aimed to assess and describe the frequency of reported use of RWE for clinical efficacy regulatory evaluation of authorised targeted therapies for the treatment of patients with solid malignancies in Europe. For that, similar use of RWE in haematological malignancies was not within the scope of this evaluation. We interpreted and classified the RWE’s role for regulatory decision based on how it was described in the EMA’s European Public Assessment Reports (EPARs), and assessed the characteristics and risk of bias (RoB) of the published RWE studies reported in the EPARs.

## Methods

### Study design and eligibility criteria

This is a cross-sectional study of all consecutive Initial Marketing Authorisations (IMA) and Extensions of Indication (EoI) granted by EMA to oncology medicines, fulfilling the following inclusion criteria: (i) targeted therapy, as per National Cancer Institute’s definition, i.e. small molecule drugs and monoclonal antibodies (not including endocrine therapy nor immunotherapy medicines); (ii) decision date for IMA or EoI between 1 January 2018 and 31 December 2022; (iii) indication for the treatment of patients with solid malignancies. The exclusion criteria are detailed in Supplementary Figure S1, available at https://doi.org/10.1016/j.esmorw.2024.100039.

### Data sources and sample selection

Since 2004, EMA provides a publicly available document for every approved medicine indication in the EU that compiles a complete overview of the assessment procedure—the EPAR.^17^^,^^18^ We extracted the Excel® listing of EPARs from EMA’s main website (hereafter referred to as EMA-database),^19^ and used it for identifying all the eligible authorised indications (Supplementary Table S1, available at https://doi.org/10.1016/j.esmorw.2024.100039). The EMA-database was last accessed on 10 March 2023.

### Data extraction of characteristics from included authorised indications

We extracted the following characteristics of the included authorised indications: (i) from EMA-database: medicine name, regulatory setting (IMA versus EoI), therapeutic area, disease setting, additional monitoring, generic, biosimilar, conditional approval, exceptional circumstances, accelerated assessment, orphan medicine, and IMA date; and (ii) from EPARs: date of opinion for EoI, authorised indication, and reported use of RWE as defined below (yes/no). In addition, each medicine was classified by a medical oncologist (DMB and AV) according to the following variables: first drug in class, targeted therapy subtype (small molecule drug, monoclonal antibody, and antibody–drug conjugate).

### EPAR screening for reported use of real-world evidence

The EPARs of all eligible indications were screened for reported use of RWE for clinical efficacy evaluation. Each EPAR was screened using a standardised approach (Supplementary Table S2, available at https://doi.org/10.1016/j.esmorw.2024.100039) by one team member (JWGD, DMB, SN, AP, AV). To validate the accuracy of the methodology, a subset of 10% of the EPARs was screened by another team member. Discrepancies were discussed and if needed shared with the team for harmonisation. We considered RWD/RWE definitions as per Flynn et al.^12^ and ESMO Guidance for Reporting Oncology real-World evidence (ESMO-GROW)^20^ (Supplementary Table S3, available at https://doi.org/10.1016/j.esmorw.2024.100039).

### Interpretation and classification of real-world evidence’s role

In case of reported use of RWE for clinical efficacy evaluation in the EPAR, we classified if the RWE studies were reported to be submitted as main study or complementary evidence, or ‘other’ if reported in other sections of the EPAR. We then interpreted EMA’s assessment of the RWE reported in the EPAR for clinical efficacy evaluation and we classified the RWE’s role as: (i) ‘definitive’ if we identified an RWE study reported to be submitted as main study or if this submitted main study contained RWD (e.g. RWD used as synthetic control arm of the main interventional trial available), (ii) ‘supportive’ when RWE was reported to be submitted as complementary evidence or was identified in another section of the EPAR, and provided indirect external comparison (e.g. historical RWD contextualizing the prognosis of the disease with standard of care), direct comparison [e.g. RWD used as synthetic control arm of complementary interventional trial(s)], or clinical effectiveness confirming the positive evidence from the main study, or (iii) ‘non-supportive’ whenever we interpreted that the reported RWE was considered of insufficient quality/relevance to support the authorisation, or provided contradictory evidence compared with the main positive evidence. Examples of this classification are reported in Supplementary Table S4, available at https://doi.org/10.1016/j.esmorw.2024.100039.

### Data extraction of characteristics from reported real-world evidence studies

We extracted the number of RWE studies per EPAR, the primary tumour type, and the following RWE study characteristics from the retrieved full publications and conference proceedings: type of research, study design, key eligibility criteria, geography, number of centres, sample size, comparator, primary and secondary endpoint, endpoint used by EMA, RWD source, and funding.

### Risk of bias assessment of published real-world evidence studies

The RoB assessment of the identified full publications was independently carried out by two investigators (DMB and AV) applying the following tools: (i) Risk Of Bias In Non-randomised Studies-of Interventions (ROBINS-I) tool for studies assessing effectiveness of medicines^21^; (ii) Quality in Prognostic Studies (QUIPS) tool for studies of prognostic factors;^22^ (iii) Newcastle-Ottawa Scale (NOS) for general epidemiological studies not fitting the two previous categories of studies.^23^ Interpretation and rating of domain-level and overall RoB as low, moderate, and serious or high risk for ROBINS-I and QUIPS were defined as per the source references of each tool.^21^^,^^22^ NOS overall score ranges from zero to nine stars, and it results from the sum of the stars attributed to each item of the scale (maximum one star for each item of selection and outcome domains, and two stars for the comparability item).^23^ The RoB assessment was harmonised using a consensus method between both investigators and validated by a third investigator (AA).

### Statistical analysis

The characteristics of the included authorised indications were described with frequencies and percentages. Fisher’s exact test was used to compare their distribution in EPARs with versus without reported use of RWE for clinical efficacy, considering a two-sided statistical significance level of 5%. Subgroup analyses were carried out for characteristics that included five or more authorised indications. Descriptive analyses were carried out using SPSS® version 27.

## Results

### Characteristics of the included authorised indications

From 1976 medicines in the EMA-database, we identified 55 medicines and a total of 75 authorised indications of targeted therapies between 2018 and 2022 for the treatment of patients with solid malignancies (Supplementary Figure S1, available at https://doi.org/10.1016/j.esmorw.2024.100039). The most common therapeutic areas were lung (21.3%, 16 of 75) and breast (20.0%, 15 of 75) cancer, and the disease setting was mostly advanced (89.3%, 67 of 75). Fifteen authorisations received conditional approval (20.0%). The number of included authorisations per year ranged from 10 (2019) to 20 (2018) (Table 1).

**Table 1:** Characteristics of the authorised indications of oncology targeted therapies between 2018 and 2022 for the treatment of patients with solid malignancies

Characteristic, *n* (% per column^a^)	Total, *n* = 75	EPARs without reported RWE, *n* = 59	EPARs with reported RWE, *n* = 16	*P* value
Therapeutic area				
Lung	16 (21.3)	10 (16.9)	6 (37.5)	0.428
Breast	15 (20.0)	10 (16.9)	5 (31.3)	
Gastrointestinal	9 (12.0)	7 (11.9)	2 (12.5)	
Gynaecological	6 (8.0)	6 (10.2)	0 (0.0)	
Genitourinary	6 (8.0)	5 (8.5)	1 (6.3)	
Melanoma	4 (5.3)	4 (6.8)	0 (0.0)	
Endocrine	2 (2.7)	2 (3.4)	0 (0.0)	
More than one^b^	17 (22.7)	15 (25.4)	2 (12.5)	
Disease setting				
Early	8 (10.7)	7 (11.9)	1 (6.3)	0.871
Early and advanced	6 (8.0)	5 (8.5)	1 (6.3)	
Advanced	61 (81.3)	47 (79.7)	14 (87.5)	
Targeted therapy subtype				
Small molecule drug	50 (66.7)	40 (67.8)	10 (62.5)	0.106
Monoclonal antibody	20 (26.7)	17 (28.8)	3 (18.8)	
Antibody–drug conjugate	5 (6.7)	2 (3.3)	3 (18.8)	
Regulatory setting				
Initial marketing authorisation	42 (56.0)	30 (50.8)	12 (75.0)	0.098
Extension of indication	33 (44.0)	29 (49.2)	4 (25.0)	
First drug in class (Yes)	19 (25.3)	14 (21.6)	5 (31.3)	0.533
Generic (Yes)	3 (4.0)	3 (5.9)	0 (0.0)	0.547
Biosimilar (Yes)	13 (17.3)	13 (23.5)	0 (0.0)	0.058
Accelerated assessment (Yes)	9 (12.0)	8 (7.8)	1 (6.3)	0.674
Orphan medicine (Yes)	4 (5.3)	3 (5.9)	1 (6.3)	1.000
Conditional approval (Yes)	15 (20.0)	8 (15.7)	7 (43.8)	*0.013*
Additional monitoring (Yes)	50 (66.7)	37 (62.7)	13 (81.3)	0.235
Year				
2018	20 (26.7)	17 (28.8)	3 (18.8)	0.608
2019	10 (13.3)	9 (15.3)	1 (6.3)	
2020	13 (17.3)	10 (16.9)	3 (18.8)	
2021	18 (24.0)	14 (23.7)	4 (25.0)	
2022	14 (18.7)	9 (15.3)	5 (31.3)	

Italics indicates a statistically significant difference of the relevant characteristic between EPARs with versus without reported RWE.

EPAR, European Public Assessment Report; RWE, real-word evidence.

a Between brackets, percentages of characteristic per column.

b Authorisations covering more than one therapeutic area in the same approval (13 biosimilars: trastuzumab and bevacizumab; 2 generics: sunitinib and imatinib; 2 tissue-agnostic: entrectinib and larotrectinib).

### Reported use of real-world evidence for clinical efficacy regulatory evaluation

The use of RWE for clinical efficacy evaluation of targeted therapies for solid malignancies was reported in the EPARs of 21.3% (16 of 75) of the included authorised indications. EPARs with reported use of RWE for clinical efficacy evaluation were more likely from indications with conditional approval, compared with EPARs without (43.8%, 7 of 16, versus 15.7%, 8 of 59, *P* = 0.013) (Table 1). In the subgroup analyses, the reported use of RWE was numerically higher in the EPARs of: (i) authorised indications for the treatment of lung (37.5%, 6 of 16) and breast (33.3%, 5 of 15) cancer versus other therapeutic areas; (ii) antibody–drug conjugates (60%, 3 of 5) versus small molecule drugs (20%, 10 of 50) and monoclonal antibodies (15%, 3 of 20); IMA (28.6%, 12 of 42) versus EoI (12.1%, 4 of 33); and authorisations with conditional approval (46.7%, 7 of 15) versus regular approval (15.0%, 9 of 60). There was a trend towards an increasing reported use of RWE for clinical efficacy regulatory evaluation over the 5 years of the study (15.0% in 2018 to 35.7% in 2022) (Supplementary Figure S2, available at https://doi.org/10.1016/j.esmorw.2024.100039).

### Role of real-world evidence for clinical efficacy regulatory evaluation

**Figure 1:**
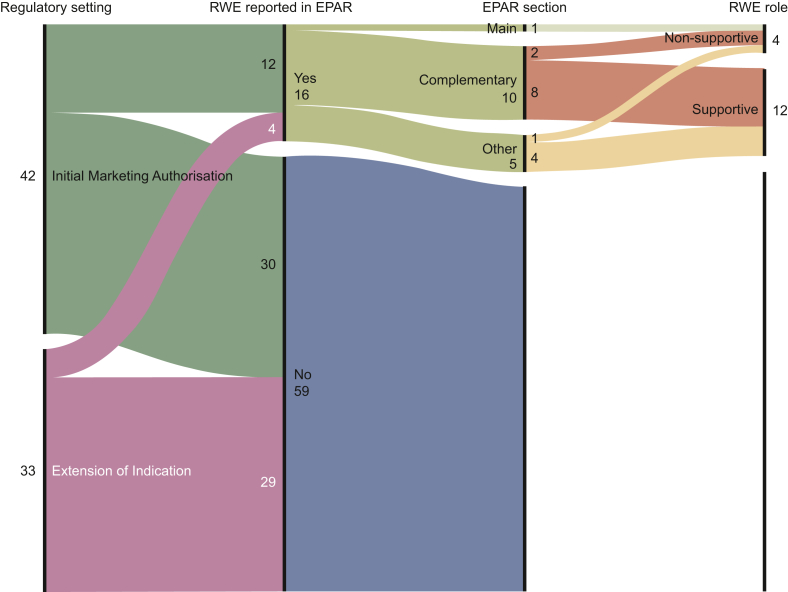
**Graphical representation of the authorised indications of oncology targeted therapies between 2018 and 2022 for the treatment of patients with solid malignancies, with illustration of the reported use of real-world evidence for clinical efficacy evaluation in the European Public Assessment Report (EPAR)**. Number of authorisations per regulatory setting (Initial Marketing Authorisation versus Extension of Indication), reported use of real-world evidence (RWE) for clinical efficacy evaluation in the EPAR (yes versus no), EPAR section where RWE was reported (main versus complementary versus other), and classification of RWE role based on how it was described in the EPAR (definitive, supportive versus non-supportive). To read from left to right, the numbers refer to the number of authorised indications per category of the graphic.

From the 16 EPARs with reported use of RWE for clinical efficacy evaluation, RWE was reported to be submitted as main study in 1, as complementary study in 10, and it was identified in other EPAR sections in the remaining 5 cases (Table 2). We classified the RWE role, based on how it was described in the EPAR, as ‘definitive’ in none, ‘supportive’ of favourable clinical efficacy evaluation in 12, and ‘non-supportive’ in 4 cases (Table 2, examples in Supplementary Table S4, available at https://doi.org/10.1016/j.esmorw.2024.100039). Ultimately, we classified RWE as ‘supportive’ source of confirmatory evidence in 12 of 16 (75.0%) EPARs with reported use of RWE for clinical efficacy evaluation, of which most were reported to be submitted as complementary evidence (66.7%, 8 of 12) (Figure 1). Authorised indications with reported use of RWE for clinical efficacy evaluation in the EPAR are described in Table 2.

**Table 2:** List of authorised indications with reported use of real-world evidence (RWE) for clinical efficacy evaluation in the European Public Assessment Report (*n* = 16), with RWE role classified as supportive (*n* = 12) or non-supportive (*n* = 4)

Medicine	Regulatory setting	Primary tumour type	Disease setting	Target therapy subtype	Target	EPAR section where RWE was reported	Number of studies^a^	Full publication references^b^
**Indications with RWE studies classified as ‘supportive’ (*****N*** **= 12)**								
Avapritinib	IMA^c^^,^^d^^,^^e^	GIST	Advanced	Small molecule	KIT-PDGFRA	Complementary study	1	^26^
Brigatinib	IMA^c^	NSCLC	Advanced	Small molecule	ALK	Complementary study	1	
Entrectinib	IMA^c^^,^^d^	More than one	Advanced	Small molecule	ROS1, ALK, TRK	Complementary study	1	^35^
Larotrectinib	IMA^c^^,^^d^	More than one	Advanced	Small molecule	TRK	Complementary study	1	
Sacituzumab govitecan	IMA^c^	Breast	Advanced	ADC	TROP-2	Other section	2^f^	^31^
Sotorasib	IMA^c^^,^^d^	NSCLC	Advanced	Small molecule	KRAS	Other section	1	
Tepotinib	IMA^c^	NSCLC	Advanced	Small molecule	MET	Complementary study	1	
Trastuzumab	IMA^c^^,^^g^	More than one	Early and advanced	Monoclonal antibody	HER2	Other section	2^f^	^32^^,^^33^
Trastuzumab deruxtecan	IMA^c^^,^^d^	Breast	Advanced	ADC	HER2	Complementary study	1	
Cabozantinib	EoI^h^	RCC	Advanced	Small molecule	VEGF, MET	Other section	1	^34^
Olaparib	EoI	Breast	Early	Small molecule	PARP	Complementary studies	3^i^	^27^^,^^28^
Ramucirumab	EoI	NSCLC	Advanced	Monoclonal antibody	VEGF	Complementary studies	2^f^	^29^^,^^30^
**Indications with RWE studies classified only as ‘non-supportive’ (*****N*** **= 4)**								
Abemaciclib	IMA^c^	Breast	Advanced	Small molecule	CDK4/6	Main study	1	^24^
Amivantamab	IMA^c^^,^^d^	NSCLC	Advanced	Monoclonal antibody	EGFR-MET	Complementary study	1	^25^
Capmatinib	IMA^c^	NSCLC	Advanced	Small molecule	MET	Complementary studies	2^j^	
Trastuzumab deruxtecan	EoI^c^^,^^d^	Gastric	Advanced	ADC	HER2	Other section	1	

ADC, antibody–drug conjugate; ALK, anaplastic lymphoma kinase; CDK4/6, cyclin-dependent kinase 4/6; EGFR, epidermal growth factor receptor; EoI, Extension Of Indication; EPAR, European Public Assessment Report; GIST, gastrointestinal stromal tumour; HCC, hepatocellular carcinoma; HER2, human epidermal growth factor receptor 2; IMA, Initial Marketing Authorisation; KIT, proto-oncogene receptor tyrosine kinase; KRAS, Kirsten rat sarcoma virus; MET, mesenchymal epithelial transition; NSCLC, non-small-cell lung cancer; PARP, poly(adenosine diphosphate–ribose) polymerase; PDGFRA, platelet-derived growth factor receptor α; RCC, renal cell carcinoma; ROS1, c-ros oncogene 1; RWE, real-world evidence; TRK, tropomyosin receptor kinase; TROP-2, trophoblast cell-surface antigen 2; VEGF, vascular endothelial growth factor.

a The column ‘Number of studies’ includes the total number of RWE studies reported in the EPAR as used for clinical efficacy evaluation.

b The number of ‘Full publication references’ cited in the EPAR may be lower than the number of studies.

c Additional monitoring.

d Conditional approval.

e Orphan medicine.

f Both studies were classified as ‘supportive’.

g Biosimilar.

h Accelerated assessment.

i Two studies were classified as ‘supportive’ and one as ‘non-supportive’.

j Both studies were classified as ‘non-supportive’.

### Characteristics of the published real-world evidence studies

We retrieved 14 publications (12 full publications^24^, ^25^, ^26^, ^27^, ^28^, ^29^, ^30^, ^31^, ^32^, ^33^, ^34^, ^35^ and 2 conference proceedings^36^^,^^37^) from the 22 RWE studies reported in the 16 EPARs with reported use of RWE for clinical efficacy evaluation (Table 2). As per ESMO-GROW definitions^20^ the type of research was mostly analytical (57.1%, 8 of 14), four studies assessing comparative effectiveness and four studies assessing prognostic or predictive factors, often using a retrospective cohort study design (57.1%, 8 of 14). Only one study was international and 11 of 14 (78.6%) originated from the United States. In most cases, the endpoint considered by EMA was the primary endpoint of the study (10 of 14), which was mostly overall survival (7 of 10). Most commonly, RWD originated from company-aggregated data sources (5 of 14) and studies were industry funded (6 of 14), which was the case for all three RWE studies classified as ‘non-supportive’ (Table 3). In the seven studies assessing effectiveness of medicines, six were classified as having serious RoB, mostly due to RoB in outcome measurement domain. Regarding the three studies assessing prognostic factors, RoB was classified as high in study attrition domain for all and in study confounding for two. Finally, for the two epidemiological studies not fitting the previous categories, the NOS scored seven and six stars. The RoB scores by specific domain, medicine name, type of study, and corresponding tool are presented in Table 4. Detailed assessments are provided as Supplementary Material, available at https://doi.org/10.1016/j.esmorw.2024.100039.

**Table 3:** Characteristics of real-world evidence studies with full publications and conference proceedings reported in the EPAR for clinical efficacy evaluation (*n* = 14)

Medicine name	Reference	Type of research (objective)	Study design	Key eligibility criteria	Geography	Centres, *n*	Sample size, *n* (TT)	Comparator	Primary endpoint	Secondary endpoint	Endpoint used by EMA	Real-world data source	Funding
Abemaciclib	Rugo et al., 2020 ^24^^a^	Analytical (comparative effectiveness)	Quasi-experimental (RWD as control)	ER+/HER2− MBC, +2L	National (USA)	N/A	281 (0)	Capecitabine Gemcitabine Eribulin Vinorelbine	OS	DoT	OS	Company-aggregated data: Flatiron Health®	Industry
Amivantamab	Bazhenova et al., 2021^25^^a^	Analytical (prognostic biomarker)	Retrospective cohort	Advanced NSCLC	National (USA)	280	3014 (2825)	None	OS	PFS	OS	Company-aggregated data: Flatiron Health®	Industry
Avapritinib	von Mehren et al., 2021^26^	Analytical (comparative effectiveness)	Quasi-experimental (RWD as control)	GIST *PDGFRA* D842Vmut Prior TKI in advanced setting	National (USA)	3	19 (19)	Not reported	OS	PFS	OS and PFS	Health records	Industry
Olaparib	Mittendorf et al., 2011^27^	Analytical (prognostic score)	Prospective cohort	EBC in neoadjuvant setting	National (USA)	1	969 (N/A)	None	BCSS	None	OS and DFS	Standardised cohort data	Academic
Olaparib	Abdelsattar et al., 2016^28^	Analytical (prognostic score)	Retrospective cohort	EBC in neoadjuvant setting	National (USA)	1	769 (N/A)	None	BCSS	None	OS and DFS	Investigator-aggregated data: centre databases and medical records	No funding
Olaparib	Batalini et al., 2021^36^^a^^,^^b^	Analytical (predictive biomarker)	Retrospective cohort	MBC with g*BRCA* or non-g*BRCA*	National (USA)	280	62 (62)	None	OS	PFS	OS and DFS	Company-aggregated health data: Flatiron Health®	Industry
Ramucirumab	Schuette et al., 2015^29^	Descriptive (treatment patterns)	Prospective cohort	Advanced NSCLC (stage IIIb/IV), 1L	National (Germany)	149	4200 (546)	None	Characteristics associated with EGFRmut	Treatment patterns	N/A	Standardised cohort data	Not reported
Ramucirumab	Li et al., 2019^30^	Descriptive (treatment and outcome patterns)	Retrospective cohort	Advanced *EGFR*mut (exon 19/21) NSCLC (stage IIIb/IV)	National (USA)	265	961 (785)	None	TNT	OS	N/A	Company-aggregated health data: Flatiron Health®	Industry
Sacituzumab govitecan	Dawood et al., 2009^31^	Descriptive (outcome patterns)	Retrospective cohort	Stage I-III TNBC	National (USA)	1	679 (N/A)	None	Proportion of patients developing BM	OS	OS	Patient/disease registry	Private not for profit
Sacituzumab govitecan	Heitz et al., 2008^37^^b^	Descriptive (outcome patterns)	Retrospective cohort	Stage I-IV breast cancer	National (Germany)	1	3193 (N/A)	None	Proportion of patients developing BM	PFS and OS	OS	Patient/disease registry	Not reported
Trastuzumab	Pernas et al., 2012^32^	Descriptive (effectiveness)	Prospective cohort	Stage II-III HER2+ breast cancer	National (Spain)	1	83 (83)	None	pCR	Safety	pCR	Health records	Not reported
Trastuzumab	Bayraktar et al., 2012^33^	Analytical (comparative effectiveness)	Retrospective cohort	HER2+ breast cancer, neoadjuvant setting	National (USA)	1	300 (292)	PH-FECH versus TCH	pCR	ORR, RFS, and OS	pCR	Patient/disease registry	Mixed (governmental and private)
Cabozantinib	Martínez Chanzá et al., 2019^34^	Descriptive (effectiveness)	Retrospective cohort	Metastatic non-clear-cell RCC	International (USA and Belgium)	22	112 (112)	None	ORR, TTF, and OS	PFS, failure at 6/12 months, safety	‘Efficacy’	Health records	No funding
Entrectinib	Tremblay et al., 2022^35^	Analytical (comparative effectiveness)	Quasi-experimental	Advanced *ROS1*mut NSCLC	National (USA)	N/A	107 (107)	Crizotinib	TTD	PFS, OS	TTD	Company-aggregated health data: Flatiron Health®	Industry

BCSS, breast cancer-specific survival; BM, brain metastases; DFS, disease-free free survival; DoT, duration of treatment; EBC, early breast cancer; EGFR, epidermal growth factor receptor; EMA, European Medicines Agency; ER, estrogen receptor; gBRCA, breast cancer gene germline mutation; GIST, gastrointestinal stromal tumour; HER2, human epidermal growth factor receptor 2; MBC, metastatic breast cancer; mut, mutated; N/A, not available; NSCLC, non-small-cell lung cancer; ORR, overall response rate; OS, overall survival; pCR, pathologic complete response; PDGFRA, platelet-derived growth factor receptor α; PFS, progression-free survival; PH-FECH, sequential paclitaxel and trastuzumab followed by FEC-75 in combination with trastuzumab; RCC, renal cell carcinoma; RFS, recurrence-free survival; RWD, real-world data; TCH, docetaxel, carboplatin, and trastuzumab; TKI, tyrosine kinase inhibitor; TNBC, triple-negative breast cancer; TNT, time to next treatment; TT, targeted therapy; TTD, time to treatment discontinuation; TTF, time to treatment failure; 1L, first-line; +2L, second-line or later.

a Study classified as ‘non-supportive’.

b Conference proceeding.

**Table 4:** Risk of bias appraisal of full publications (*n* = 12), stratified by study type and corresponding tool, including ROBINS-I, QUIPS, and NOS

Risk of bias in non-randomised studies of interventions (ROBINS-I) tool for studies assessing effectiveness of medicines									
Medicine name	Author, year	Bias due to confounding	Bias in selection of participants into the study	Bias in classification of interventions	Bias due to deviations from intended interventions	Bias due to missing data	Bias in measurement of outcomes	Bias in selection of the reported result	Overall bias
Trastuzumab	Bayraktar, 2012^33^	Moderate	Low	Moderate	Serious	Moderate	Moderate	Low	Serious
Cabozantinib	Martínez Chanzá, 2019^34^	Low	Low	Low	Low	Moderate	Serious	Low	Serious
Ramucirumab	Li, 2019^30^	Serious	Low	Moderate	Low	Moderate	Serious	Low	Serious
Avapritinib	von Mehren, 2021^26^	Moderate	Moderate	Moderate	Low	Moderate	Serious	Low	Serious
Trastuzumab	Pernas, 2012^32^	Moderate	Low	Low	Low	Low	Moderate	Low	Moderate
Abemaciclib	Rugo, 2020^24^^a^	Moderate	Low	Low	Moderate	Serious	Serious	Low	Serious
Entrectinib	Tremblay, 2022^35^	Moderate	Low	Low	Moderate	Serious	Serious	Moderate	Serious

Quality in Prognostic Studies (QUIPS) tool for studies of prognostic factors							
Medicine name	Author, year	Study Participation	Study Attrition	Prognostic Factor Measurement	Outcome Measurement	Study Confounding	Statistical Analysis and Reporting
Olaparib	Abdelsattar, 2016^28^	Low	High	Low	Low	High	Moderate
Amivantamab	Bazhenova, 2021^25^^a^	Low	High	Moderate	Moderate	Low	Low
Olaparib	Mittendorf, 2011^27^	Low	High	Low	Low	High	Moderate

The Newcastle-Ottawa Scale (NOS) for general epidemiological studies not fitting the two previous categories of studies				
Medicine name	Author, year	Selection	Comparability	Outcome
Ramucirumab	Schuette, 2015^29^	✵✵✵✵	N/A	✵✵✵
Sacituzumab govitecan	Dawood, 2009^31^	✵✵✵	✵✵	✵

Stars indicate the NOS overall score which ranges from zero to nine stars, and it results from the sum of the stars attributed to each item of the scale.

a Studies classified as ‘non-supportive’.

## Discussion

Our study describes the reported use of RWE for clinical efficacy evaluation in the EPARs of 16 out of a total of 75 consecutive authorised indications of targeted therapies for the treatment of patients with solid malignancies between 2018 and 2022. We classified RWE as ‘supportive’ confirmatory evidence in 12 of 16 indications, and most of these RWE studies were reported to be submitted as a complementary source of evidence. Overall, therapeutic areas in which RWE was most frequently reported included lung and breast cancer, which might relate to the higher prevalence of these malignancies, with more research and many targeted therapies recently approved for these tumour types. We also found that clinical efficacy evaluations reporting RWE more often resulted in conditional marketing authorisation for the indication. Hence, when lower level of evidence than normally required is available for benefit–risk assessment (e.g. main study is a non-randomised single-arm clinical trial), authorities may grant conditional approval, and more likely consider RWE under the premisses that the applicant should be in a position to provide more robust clinical data in the future. Interestingly, we observed an increasing trend for the use of RWE related to clinical efficacy evaluation of authorised indications of targeted therapies for the treatment of patients with solid malignancies over time from 2018 to 2022. This suggests a growing importance of this type of evidence for future-proofing of the regulatory approval system.

Over the past years, several studies on RWE incorporation in FDA and EMA applications reported that the use of RWE for demonstrating safety and efficacy was highest for oncology.^10^, ^11^, ^12^ A previous study that focussed on oncology medicines reported that 38% of early development, 58% of clinical development, and 63% of registration decision sections of the selected EPARs contained RWE.^14^ This overall higher presence of RWE can be explained by the fact that our study was focused only on RWE related to the efficacy of targeted therapies for solid malignancies, which in most cases is still scarce before having regulatory authorisation. Regarding studies that more specifically investigated the role of clinical efficacy, Arondekar et al.^13^ previously assessed the role of RWE in clinical efficacy evaluation among 133 original FDA approvals for oncology medicines in the United States between 2015 and 2020 and found that only 11 (8%) applications included RWE. The setting of the latter is more comparable to our study, and the lower frequency of RWE (8% versus 21%) might relate to the fact that (i) we are focusing on targeted therapies, frequently developed for smaller subgroups where RCTs are more difficult to conduct, and (ii) FDA typically precedes EMA’s approvals, thus allowing RWE to be generated in the United States before European authorisations take place.^38^ Indeed, in our study, we found that 11 of the 14 publications of RWE cited in the EPARs originated from the United States. Lastly, a third study by Lau et al.^39^ reported higher usage of RWE by FDA and EMA on oncology products approved in 2020 and 2021. The percentages of submissions with RWE/historical reviews conducted by Health Canada, FDA, and EMA were reported to be 24%, 76%, and 56%, respectively. These higher rates of RWE usage for oncology approvals compared with our results can be explained by our focus on effectiveness only, and possibly because during that period a high percentage of targeted therapies were approved for haematological malignancies that used RWE as support for authorisation which were not included in our study.

Further evaluation of publications of RWE studies cited in the EPARs demonstrated that most were analytical, i.e. designed to quantify a relationship or association between different variables—exposures and outcomes (including comparative effectiveness and prognostic/predictive factors), non-international, and retrospective cohort studies, mimicking the findings of a systematic review of our ESMO working group.^6^ In most cases, the primary endpoint of the RWE studies included in our analysis (mostly overall survival) was the same as the main endpoint used by EMA for regulatory deliberations. This shows an appropriate selection of RWE studies focusing on a clinically relevant outcome of interest. Lastly, pharmaceutical companies, as applicants, may prioritise studies in which they are involved in both funding and data collection since most studies used company-aggregated health data sources and were industry funded. Another potential reason for this finding could be the lack of large-scale, investigator-initiated, international RWE studies,^6^ which could be tackled by facilitated access to these company-aggregated databases for academic research purposes.

Notably, we found a high prevalence of important RoB in published RWE studies cited within the scope of the assessed EPARs. That is aligned with a recent study in which the authors concluded that the quality of contemporary oncology RWD studies is often of insufficient quality to trustfully inform regulatory bodies and clinical practice.^40^ Moreover, the authors advocated that (i) investments in high-quality RWD are needed to improve RWE, and (ii) that quality assessment tools can facilitate improvements in the design, execution, and reporting of RWE studies when applied before submission for publication. Recently, the unmet need for guidance in reporting RWE studies specifically for oncology was fulfilled by the development of the ‘ESMO-GROW’.^20^ This guidance includes detailed key recommendations that incorporate several peculiarities of modern RWE research in oncology and specify what should be reported and how.

The main limitation of our study relates to the data source. Firstly, detailed outlines of the literature discussed by EMA’s Committee for Medicinal Products for Human Use (CHMP) are not always systematically captured in the EPARs, including inaccurate or lack of citations of the RWE studies discussed. In addition, since a dedicated paragraph for RWE is not present in the EPAR, RWE can be described in multiple sections and the document may not necessarily include all RWE studies submitted by the applicant and assessed by EMA. This limitation might lead to an underestimation of RWE use in clinical efficacy evaluation and affect our interpretation of the reported RWE’s role in regulatory decision making. As per the EMA’s robust review and reporting methodologies, however, it can be assumed that the most relevant RWE considered for each decision was included in the EPARs, thus we consider the data source reliable for the purpose of this study. Although there is no uniform approach to this type of data collection and analysis, our methodology was based on previous studies in order to facilitate study outcome comparisons.^12^^,^^15^ Moreover, to minimise inter-observer variability, we included multiple cross-validation procedures and discussions to resolve discrepancies or uncertainties among the multiple researchers until consensus was reached. In the absence of a uniform nomenclature, this process of data collection remains time-consuming and labour intensive. The oncology community needs a clear, structured, and accountable framework of RWD assessment used for regulatory decisions, and transparent reporting, for instance in the EPAR. As per the importance of this topic, the EMA is currently developing a framework to standardise the assessment and reporting procedure of RWE studies.^9^ Further work is needed for fulfilling the EU’s vision to enable the use of RWE and establish its value across regulatory use cases by 2025.^7^ Another limitation was the restriction to EPARs of authorised oncology targeted therapy indications for the treatment of patients with solid malignancies. Hence, our study does not provide insight in the degree of use of RWE in all oncology targeted therapy applications. Nevertheless, this restriction was made deliberately, as solid malignancies are the focus of the ESMO working group that conceived and developed this study, and EPAR documentation of withdrawn or refused applications is usually less extensive and could therefore have provided us with a less realistic account of RWE involvement. This study also has strengths related to the design and data collection. First, the use of publicly available data allows for reproducibility and replication of the methodology. Second, we covered a 5-year period to include a large number of therapies and to allow for investigating possible trends over time. Third, our methodology was transparent and robust given the incorporation of medicine selection in duplicate, expert validation, and cross-validation during screening and data extraction. Lastly, characterisation and RoB analysis of the included and published RWE studies provides knowledge of the quality of recently submitted evidence.

To conclude, RWE related to clinical efficacy evaluation was reported in 21% of all targeted therapy authorised indications for the treatment of patients with solid malignancies between 2018 and 2022, tending to increase over time. We classified the role of RWE submitted by the applicant as ‘supportive’ confirmatory evidence in 75% of the EPARs reporting RWE use for clinical efficacy evaluation, but we found an important RoB of the published RWE studies. Our results contribute to understanding the amount, contribution, type, and quality of RWE use in EMA oncology targeted therapy authorisations. Given the relevant and increasing use of RWE for clinical efficacy regulatory evaluation and the identified limitations of the RWE used, we highlight the need to keep strengthening global multi-stakeholder collaboration between e.g. researchers, regulators, Health Technology Assessment agencies, industry, clinicians, cancer centres, and RWE experts to increase current standards and exploit the potential of RWE in the continuous regulatory lifecycle of cancer medicines—both before and after authorisation. Future projects in collaboration with regulatory agencies such as EMA including all submitted applications for new marketing authorisation and being granted access to non-publicly available data, could provide more insight into the current value of RWE for clinical evaluation regulatory decisions in Europe.
