# Supplementary material for: Real-world evidence reported for clinical efficacy evaluation in European Public Assessment Reports of authorised targeted therapies for solid malignancies: a comprehensive review (2018-2022)
Source: ESMO Real World Data Digit Oncol. 2024 Jun 3;4:100039. doi: 10.1016/j.esmorw.2024.100039 (PMC12836655; doi:10.1016/j.esmorw.2024.100039)
Supplement: Supplementary Tables and Figures [file mmc1.docx]

**Supplementary Files**

**Supplementary Table 1.** Stepwise EMA-database screening approach.

| **Step** | **Description** |
| --- | --- |
| 1. Screening of indications and validation of medicines | The list of indications was screened for eligibility in duplicate (JWGD, DMB) from the EMA-database, and the medicines of each selected indications were validated by two senior medical oncologists (SD, GP). |
| 2. Validation of the screened indications | All the screened authorised indications from the EMA-database were validated by a third author (AP, SN, AV) on the respective EMA website page and by consulting the file “Procedural steps taken and scientific information after the authorisation” on “Assessment history” section of EMA webpage. |
| 3. Identification of additional indications | An automated search was performed consulting the file “Procedural steps taken and scientific information after the authorisation” (AP, SN, AV) for the specific key terms: “4.1” (indications section of the Summary of Product Characteristics) and “C.I.6.a.” (code for new approved indication). This search retrieved all the IMA and EoI with dates within the study period but before the decision date listed in the EMA-database. |
| 4. Retrieval of an EPAR per indication | Finally, the EPAR documents (“EPAR: Public Assessment Report” and “EPAR: Public Assessment Report - Variation”) related with each eligible indication (IMA and EoI, respectively) were identified. |

**Supplementary Table 2.** Stepwise EPAR screening approach.

| **Step** | **Description** | **Additional specification** |
| --- | --- | --- |
| 1. Automated screening of full EPAR report on pre-specified RWD/RWE keywords. | Keywords: Retrospective, Cohort, Historical, Non-randomized, Non-randomised, Cross-sectional, Case-control, Observational, Real-world, Routinely (collected data), Registry, Registries, Electronic, Health record, Emulation, Emulated, Target trial, Non-interventional, Longitudinal, Uncontrolled, Compassionate, Expanded, Early access, Post-marketing. | Composed keywords were always included both with and without the hyphen (-), and keywords were always included both with US and UK spelling. |
| 2. Detailed assessment of sections reporting use of RWE. | Read full section in which RWD/RWE reported use was identified by automated search (step 1). |  |
| 3. Screening of relevant sections for the reported use of RWD/RWE for clinical efficacy evaluation. | Sections: Clinical aspects, Clinical efficacy, Benefit-risk balance. | “Summaries of main studies” involved in these sections were included (summary or tabular overviews). |
| 4. Total reference list cross-check. | Check whether references could include RWE. | There is no specific reference section in the EPAR report, but footnotes in some pages (keywords in foot notes will also be captured by the automated search). Footnote references related to background in the “scientific discussion” section of an EPAR will not be evaluated in detail unless also referenced to in the relevant EPAR sections mentioned in step 3. |

**Supplementary Table 3.** Types of RWD/RWE included/excluded during data extraction, based on the definitions by Flynn et al. [10] and ESMO-GROW [18] ^a^.

| **Included as RWD/RWE** | **Not included as RWD/RWE** |
| --- | --- |
| Patient-based non-interventional pre-or post-authorization studies performed to support the marketing authorization application (primary and/or secondary use of data) | Non-product related literature review (for example reviews related to the natural history of the targeted disease or comorbidities associated with the disease) |
| Use of RWD source(s) (e.g., registry, electronic health care records, medical charts, etc.) to provide comparator (historical or contemporaneous) arms in single arm trials | Use of aggregated epidemiological data from multiple sources where attribution is unclear |
| Use of RWD source(s) in the context of RCTs (e.g., to assess the representativeness of the control arm) | Interventional studies (phase I, II), including pre-clinical studies, toxicological studies, dose-response studies, drug-drug interaction studies |
| Use of RWD data source(s) to support the implementation of clinical trials (e.g., by facilitating identification of eligible patients or type of data  collection) | Phase III/IV studies without RWD/RWE use (e.g.,  single-arm study without comparator group from RWD) |
| Product-related literature review (e.g., on real-world safety data of the product in other indications) | Open-label follow-up studies of clinical trial patients |
| Additional pharmacovigilance activities | Routine pharmacovigilance activities |
| Data of patients in Compassionate Use Program, and data of Post-marketing experience | Active surveillance based on spontaneous reporting |
|  | Surveys not based on individual patients (e.g., surveys of physicians to assess awareness of risk minimization measures) |

a - Definitions: (i) RWD - data obtained from observational (non-interventional) methodologies; normally related to patient health status and/or the delivery of health care; can be routinely collected from various sources such as registries, EHRs, claims, patient-generated data, biomarker data and wearable devices; and (ii) RWE – studies that utilize RWD as a source for investigating the research question(s)

**Supplementary Table 4.** Examples of text fragments extracted from the European Public Assessment Report of authorised indications with reported use of real-world evidence for clinical efficacy evaluation, referring to cases in which we classified real-world evidence’s role as supportive or non-supportive of favourable regulatory decision based on how it was described in the EPARs.

| **Supportive** | **Non-supportive** |
| --- | --- |
| “*RWE study supporting* the prognostic role of CPS & EG score; *this tool is included in the approval* as a part of definition for high-risk patients” | “So, given the identified limitations and remaining uncertainties related to the use of the Unicancer cohort, *the results from this cohort cannot be considered supportive*” |
| “*Real world data show* that patients with an ECOG performance status of ≥1 *can still be treated with an EGFR inhibitor*” | “The virtual *RWD analysis* in Western subjects is *not considered sufficiently reliable nor representative* of an entire population to bridge to the Asian population” |
| “The reported *median OS for patients with brain metastasis in the control arm is in the upper range* of what was reported for TNBC patients with brain metastasis in *historical controls*” | “Indeed, all the evidence points into the *opposite direction*, but a *definitive conclusion cannot be drawn given the quality of the evidence available*” |
| “*Supportive data of relevance* to the histology-independent indication briefly mentions results of 29 patients treated in single patient protocols or *under compassionate use*” | “In conclusion, *this study does not provide valuable insight* concerning the contextualization of the results” |
| “Only patients with RCC with a clear-cell component were eligible for CA2099ER. Only a few (three) patients were documented to also have non-clear cell component. Even if patients with only non-clear cell RCC were not included in the trial, they were not excluded from the sought indication, *which is acceptable because cabozantinib has shown efficacy in non-clear cell RCC in a retrospective study*” | “In order to place the MONARCH 1 OS data in clinical context, *OS results from a retrospective cohort study using observational data* from the Flatiron Health EMR database for patients with mBC are presented. It is entirely *unclear whether this is representative* of the difference in OS that would be seen in a randomized comparative trial. In the current disease setting, with an ORR of 20%, the numerical OS difference compared to external data is considered *a critically* *uncertain evidence of clinical benefit*.” |

Note: this classification and all data extracted from EPARs reporting RWE were validated by a second investigator


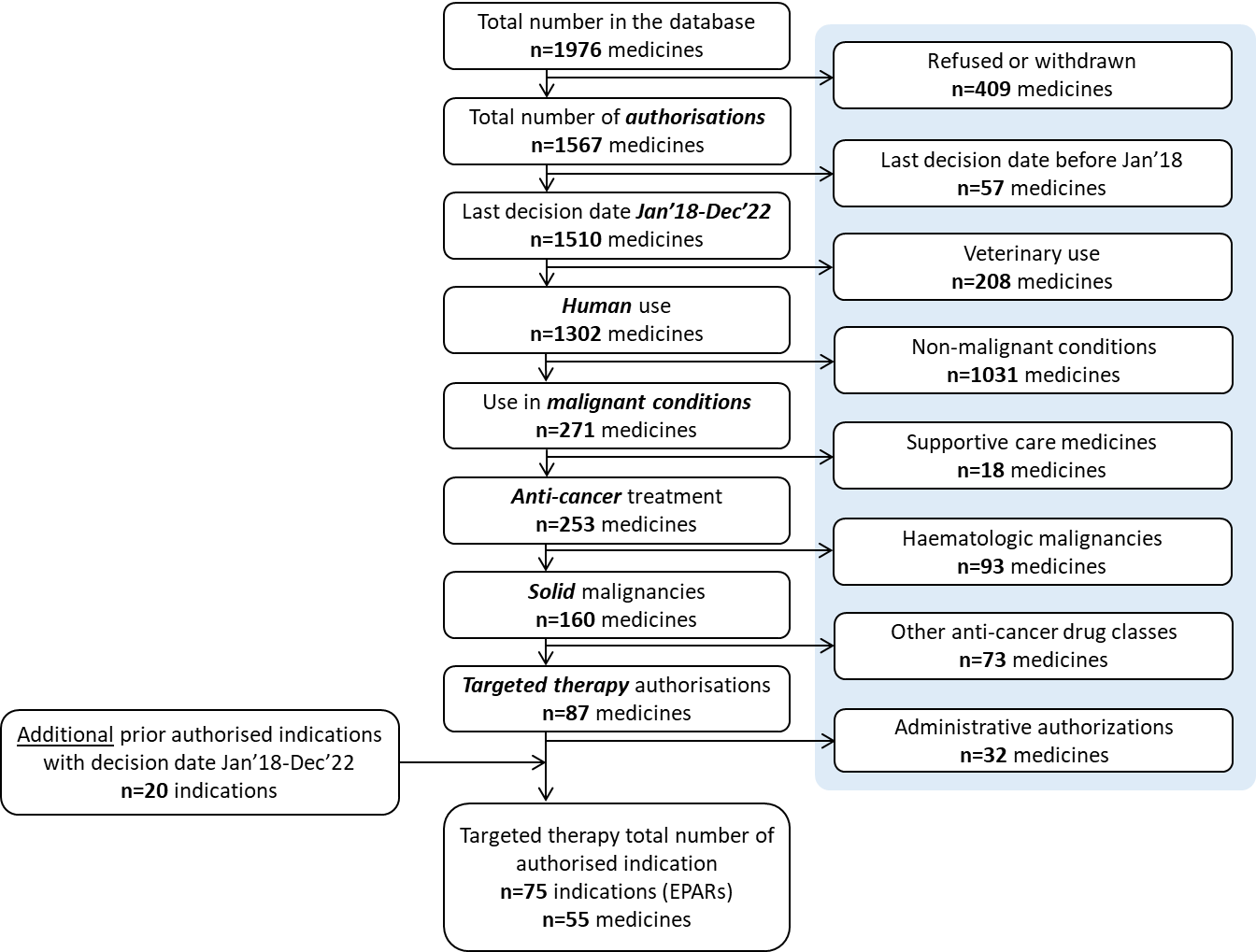


**Supplementary Figure 1.** Selection flowchart of all consecutive European Medicines Agency’s authorised indications of oncology targeted therapies between 2018 and 2022 for the treatment of patients with solid malignancies. The blue box presents the number of excluded medicines and reasons for exclusion: (i) refused/withdrawn applications; (ii) authorised indications for veterinary use; (iii) authorised indications for other non-malignant conditions; (iv) authorised indications for supportive care; (v) authorised indications for haematologic malignancies; and (vi) authorised indications for other classes of oncology medicines (e.g. chemotherapy, endocrine therapy, immune checkpoint inhibitors, cellular or gene therapies). EPARs: European Public Assessment Reports.


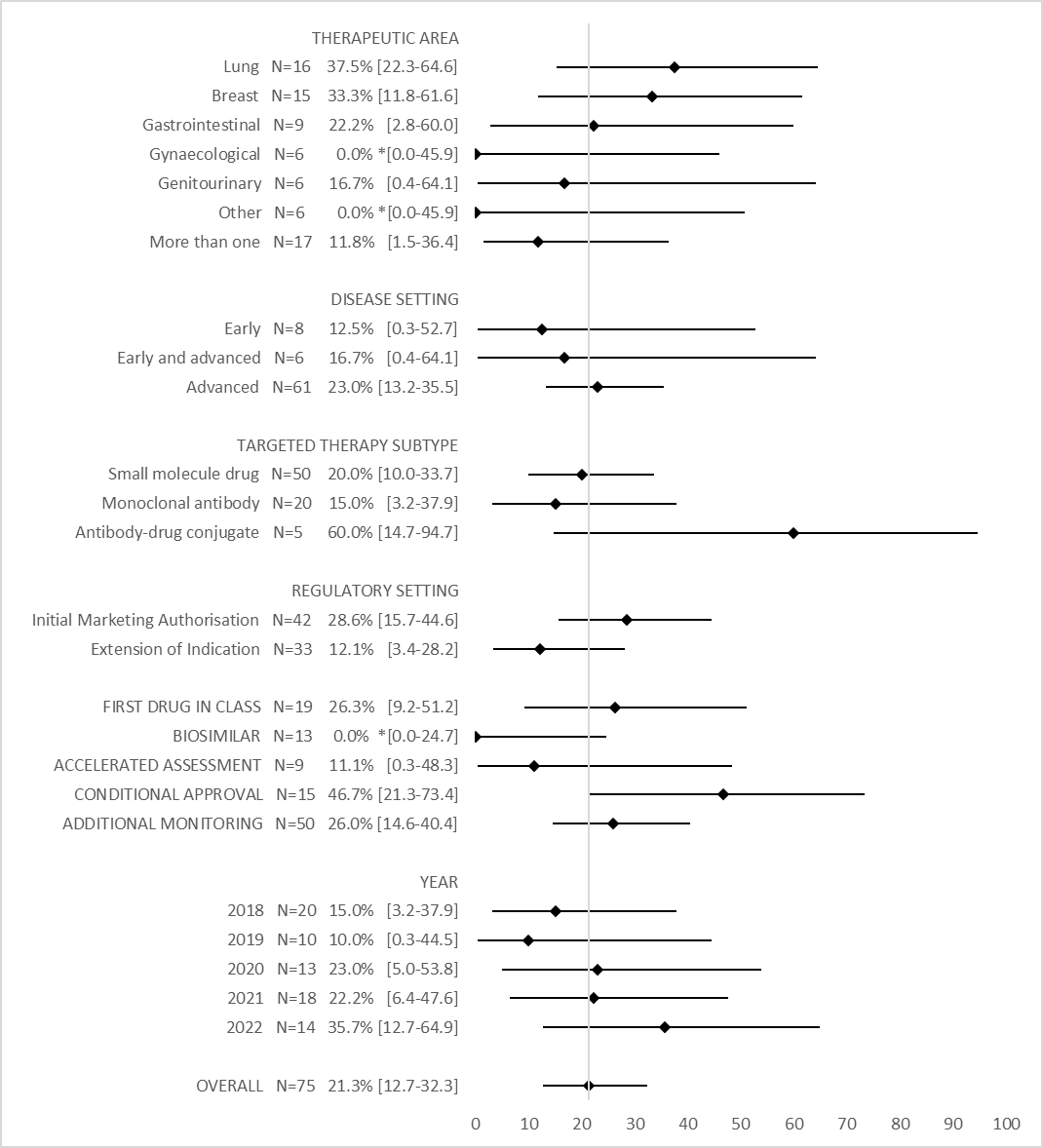


**Supplementary Figure 2.** Forest plot estimating the proportion of authorisations with real-world evidence involvement by subgroup. On the left side, we report the sample size of each subgroup, the estimated proportion of real-world evidence involvement, and the 95% confidence interval between brackets. The same data is reported at the bottom for the whole sample. Binominal “exact” test was used to estimate 95% confidence intervals. *One-sided 97.5% confidence interval.
